# Enantioseparation of Mirabegron Using Cyclodextrin‐based Chiral Columns: High‐performance Liquid Chromatography and Molecular Modeling Study

**DOI:** 10.1002/jssc.70132

**Published:** 2025-04-09

**Authors:** Ali Mhammad, Gergely Dombi, Máté Dobó, Zoltán‐István Szabó, Béla Fiser, Gergő Tóth

**Affiliations:** ^1^ Department of Pharmaceutical Chemistry Semmelweis University Budapest Hungary; ^2^ Center for Pharmacology and Drug Research & Development Semmelweis University Budapest Hungary; ^3^ George Emil Palade University of Medicine, Pharmacy Science, and Technology of Targu Mures Targu Mures Romania; ^4^ Sz‐imfidum Ltd Lunga Romania; ^5^ Institute of Chemistry University of Miskolc Miskolc Hungary; ^6^ Department of Biology and Chemistry Ferenc Rákóczi II Transcarpathian Hungarian College of Higher Education Transcarpathia Ukraine; ^7^ Department of Physical Chemistry Faculty of Chemistry University of Lodz Lodz Poland

**Keywords:** Betmiga, cyclodextrin, chiral recognition, chiral separation, mirabegron

## Abstract

A novel high‐performance liquid chromatography method for the enantioseparation of mirabegron (*R*‐mirabegron), a selective β‐3 adrenergic receptor agonist, using cyclodextrin (CD)‐based chiral stationary phases (CSPs) was developed. Seven different CSPs containing β‐, γ‐, hydroxypropyl‐β‐, sulfobutyl‐β‐, carboxymethyl‐β‐, permethyl‐β‐, and phenylcarbamate‐β‐cyclodextrin were evaluated under both polar organic and reversed‐phase conditions. Only the phenylcarbamate‐β‐cyclodextrin containing the Chiral CD‐Ph column displayed enantiorecognition. Optimization of conditions using a full factorial design led to the determination of the most suitable conditions: a mobile phase composition of 90:10:0.1 methanol:water:diethylamine, a flow rate of 0.8 mL/min, and a column temperature of 40°C with enantiomeric elution order, where the impurity *S*‐mirabegron elutes first. Using the optimized conditions, enantioseparation with *R*_s_ = 1.9 was achieved within 10 min. The developed method was validated according to current guidelines and successfully applied for the determination of *S*‐mirabegron, as a chiral impurity in pharmaceutical formulations. The enantiorecognition mechanism was investigated by molecular docking and thermodynamic analysis. Using molecular modeling, the interactions between CDs and the analyte were analyzed at the molecular level, revealing that mirabegron interacts primarily with the phenylcarbamate groups on the outer surface of the structure. Enthalpy‐controlled enantioseparation was consistently observed across all eluent compositions, regardless of the conditions. The developed and validated method is highly suitable for routine determination of the enantiomeric purity of mirabegron, offering a reliable tool for regulatory compliance.

## 1 Introduction

Many bioactive compounds contain at least one chiral center. In most cases, the pharmacological effect is attributed to only one enantiomer, referred to as the eutomer, while the other enantiomer, known as the distomer, often has minimal or no therapeutic effect and may even cause undesirable or toxic reactions [1]. Therefore, the administration of enantiopure drugs not only offers significant therapeutic advantages but is also a regulatory requirement. Regulatory bodies such as the Food and Drug Administration and the European Medicines Agency enforce stringent enantiomeric purity requirements for these drugs. For detecting low levels of enantiomeric impurity, modern, robust, and sensitive methods are essential. Several methods are available for enantioseparation, including capillary electrophoresis, gas chromatography, and high‐performance liquid chromatography (HPLC) [2]. Among these, HPLC has emerged as a highly effective technique for enantioseparation, dominating both the scientific literature and pharmaceutical compendia [3]. Direct HPLC enantioseparation using a chiral stationary phase (CSP) is considered the gold standard in the field, where the selection of the most appropriate CSP is critical for the success of enantioseparation. Hundreds of chiral columns are available on the market, such as polysaccharide‐, protein‐, crown ether‐, macrocyclic antibiotic‐, and cyclodextrin (CD)‐based CSPs [4].

CDs are widely employed as chiral selectors in electrophoretic and chromatographic techniques [5, 6, 7]. CDs are cyclic oligosaccharides, composed of α‐D‐glucopyranose units linked by α‐1,4‐glycosidic bonds, and have long been established as versatile chiral selectors. The number of glucopyranose units varies across the different types of CDs (α‐, β‐, and γ‐CD), and while natural CDs exhibit good enantiomeric resolution, their effectiveness can be significantly enhanced through derivatization. The hydroxyl groups on CDs open the way for an infinite number of derivatives and the attachment of these chiral selectors to silica gel. The use of CD‐based CSPs in reversed‐phase HPLC has contributed to advancements in enantioseparation, although polysaccharide‐based CSPs remain more widely utilized. The development, pioneered by Armstrong and DeMond in the 1980s, enabled the commercial production of CD‐based CSPs and provided a key tool for the efficient separation and analysis of enantiomers in both research and industrial applications [8]. In recent years, numerous studies have focused on the development and fabrication of modern CD‐based CSPs. Click reactions, such as ‘thiol‐ene’ and ‘azide‐alkyne’ systems, have primarily been used for immobilizing CD derivatives onto silica. For this method not only the native CDs but also differently substituted (like methylated, hydroxypropylated, or differently monosubstituted) CDs can be anchored to silica particles, resulting in a number of new CSPs [9]. The CD‐based chiral selectors containing arylcarbamate substituents (such as phenylcarbamate or naphthylcarbamate) offer promising enantioseparation performance for many compounds [10, 11]. In our previous study, we demonstrated that phenylcarbamate‐β‐CD is highly effective in separating a broad range of enantiomers under polar organic conditions, delivering significantly better performance compared to β‐CD columns [12].

**FIGURE 1:**
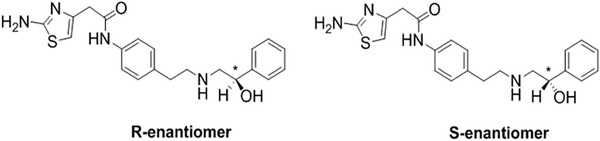
The structures of R‐mirabegron as eutomer and its antipode, *S*‐mirabegron.

Mirabegron (2‐(2‐amino‐1,3‐thiazol‐4‐yl)‐N‐[4‐[2‐[[(2R)‐2‐hydroxy‐2‐phenylethyl]amino]ethyl]phenyl]acetamide, MIR) is a selective β‐3 adrenergic receptor agonist, commonly used in the treatment of overactive bladder [13, 14]. MIR possesses a single asymmetric carbon atom, resulting in two enantiomeric forms, namely *R*‐MIR (commonly referred to as MIR) and *S*‐MIR (Figure 1). In therapy, the more effective *R*‐enantiomer is utilized. A novel monograph in European Pharmacopeia 11.2 has been released on MIR [15]. Regulatory guidelines mandate the quantification of the *S*‐enantiomer as an enantiomeric impurity, with a required detection of 0.2% relative to MIR. For enantiomeric purity analysis, the monograph specifies the use of a “beta‐cyclodextrin derivative of silica gel for chiral separation” as the stationary phase. Interestingly, however, it does not specify the exact type of β‐CD‐based column to be used, despite the numerous β‐CD‐based CSPs with different chemical structures available on the market.

A comprehensive review of the scientific literature highlights the wide variety of analytical techniques employed in the study of MIR [16, 17, 18, 19, 20, 21, 22, 23, 24, 25]. Several HPLC methods have been developed for the characterization of MIR degradation products and their quantification in dosage forms and biological fluids. Nevertheless, the number of scientific articles regarding the chiral separation of MIR is limited. Zhou et al. developed a normal‐phase method for the enantioseparation of MIR, employing a hexane‐ethanol‐based eluent on a polysaccharide‐type Chiralpak AY‐H column [26]. While this method achieved excellent separation (*R*_s_ = 3.96), it was not adopted for quality control, and the enantiomer elution order was suboptimal. Moreover, in routine quality control, less toxic and easier‐to‐apply polar organic or reversed‐phase modes are often preferred [27].

The aim of this study was to develop an efficient chiral HPLC method using CD‐based CSPs applying polar organic and reversed‐phase mode for the quality control of the single‐enantiomer drug, MIR. Our aim was to compare the different CD‐based chiral columns and select the most suitable for the baseline separation of MIR in the shortest runtime. A comprehensive analysis of chromatographic parameters influencing the separation process was also conducted using experimental design, alongside a detailed investigation of the chiral recognition mechanism by molecular modeling and thermodynamic analysis.

## 2 Materials and Methods

### 2.1 Materials

*R*‐MIR and racemic MIR were from LGC Standards GmbH. (Wesel, Germany). HPLC‐grade methanol (MeOH) and acetonitrile (ACN) were acquired from Merck KGaA (Darmstadt, Germany). High‐purity diethylamine (DEA) and triethylamine (TEA) were purchased from Sigma‐Aldrich (Budapest, Hungary). Ultrapure deionized water was prepared using a Milli‐Q Direct 8 system (Millipore, Milford, MA, USA). Chiral CD‐Ph column (250 × 4.6 mm, particle size 5 µm), Nucleodex β‐PM column (200 × 4 mm, particle size 5 µm) were ordered from Phenomenex (Torrance, CA, USA). The Astec CYCLOBOND I 2000 Chiral HPLC Column (250 × 4.6 mm, particle size 5 µm) was purchased from Sigma‐Aldrich, Hungary. Quest‐C2 (250 × 4.6 mm, particle size 5 µm), Quest‐CM (250 × 4.6 mm, particle size 5 µm), Quest‐SB (250 × 4.6 mm, particle size 5 µm), and Quest γ‐CD columns (250 × 4.6 mm, particle size 5 µm) were products of ChiroQuest Ltd. (Budapest, Hungary). Betmiga (containing 50 mg MIR, Astellas Pharma) was obtained from the Central Pharmacy of Semmelweis University (Budapest, Hungary).

### 2.2 HPLC Analysis

Method development was conducted using a JASCO HPLC system, consisting of a JASCO PU‐2089 Plus quaternary gradient pump, AS‐4050 autosampler, MD‐2010 Plus diode array detector, and CO‐2065 Plus column oven (JASCO International Co. Ltd., Tokyo, Japan). The system was operated, and data was processed using ChromNAV (ver. 2.0) software. The finalized method was subsequently transferred to an Agilent 1260 Infinity HPLC system, which included a G1312B binary gradient pump, G1367E autosampler, and G1315C diode array detector (Agilent Technologies, Waldbronn, Germany), with Agilent MassHunter (ver. 10.1 build 10.1.67) software utilized for data analysis.

The enantioseparation performance of seven distinct CD‐based HPLC columns was assessed under both reversed‐phase and polar organic chromatographic conditions: Astec Cyclobond I 2000 (β‐CD), Quest γ‐CD (γ‐CD), Nucleodex β‐PM (permethyl‐β‐CD), Quest C2 (hydroxypropyl‐β‐CD), Quest CM (carboxymethyl‐β‐CD), Quest SB (sulfobutyl‐β‐CD), and Chiral CD‐Ph (phenylcarbamate‐β‐CD). The applied mobile phase conditions are summarized in Table S1. The chromatographic parameters were maintained consistently throughout the screening process with a 0.7 mL/min flow rate and 25°C column temperature. Three replicate measurements were performed in each case. During method optimization, experimental designs were developed using Design Expert 7.0 statistical software (Stat‐Ease, Minneapolis, MN, USA). MeOH was used as the solvent for preparing all sample solutions throughout the study. For the preliminary experiments, stock solutions of 1 mg/mL MIR and 1 mg/mL racemic MIR were individually prepared in MeOH. A 150 µL aliquot of each stock solution was then combined, and the final volume was adjusted to 1 mL with MeOH. The final test solution of MIR used for method validation and applicability assessments had a concentration of 2 mg/mL. The impurity level was calculated as a percentage relative to this concentration (e.g., an impurity level of 0.1% corresponds to 2 µg of distomer per 2000 µg of MIR). An injection volume of 5 µL was used. The detection wavelength was 254 nm throughout the study.

### 2.3 Real Sample Analysis

Ten tablets of Betmiga 50 mg were accurately weighed, ground, and homogenized in a mortar. An appropriate portion of the resulting tablet powder, equivalent to approximately 50 mg of MIR, was transferred to a 25 mL volumetric flask. MeOH was added to the flask to achieve the desired volume. The suspension was then sonicated for 30 min to ensure complete dissolution. Following sonication, the sample was filtered through a 0.22 µm pore size syringe filter with a PVDF membrane (Filter Bio Membrane Co., Ltd., Nantong City, China).

### 2.4 Molecular Docking

To model the complex formation between MIR enantiomers and the specific chiral selector of the Chiral CD‐Ph, containing phenylcarbamate‐β‐CD, molecular docking calculations were carried out. Furthermore, β‐CD as an additional host was also studied. The host molecules, phenylcarbamate‐β‐CD and β‐CD were optimized during which energy minimization of the structure was carried out by combining the OPLS‐AA force field [28], and the Born implicit solvation model as it was employed before in a previous study [12].  The three‐dimensional structures of MIR enantiomers (*R*‐MIR and *S*‐MIR) were also prepared and two PDBQT files were generated. These files included all necessary information regarding the guest structures and were used as inputs during the molecular docking calculations [29]. AutoDock Vina was employed to carry out the calculations and determine the binding affinity of MIR enantiomers toward phenylcarbamate‐β‐CD and β‐CD. The calculations were carried out by setting the exhaustiveness to 8, while a 30 × 30 × 30 Å^3^ grid box was considered. For both *R*‐ and *S*‐MIR a maximum of nine binding modes were determined. The binding modes with the lowest binding affinities (*E*_A_) were selected and compared.

## 3 Results and Discussion

### 3.1 Chiral Stationary Phase Screening

**FIGURE 2:**
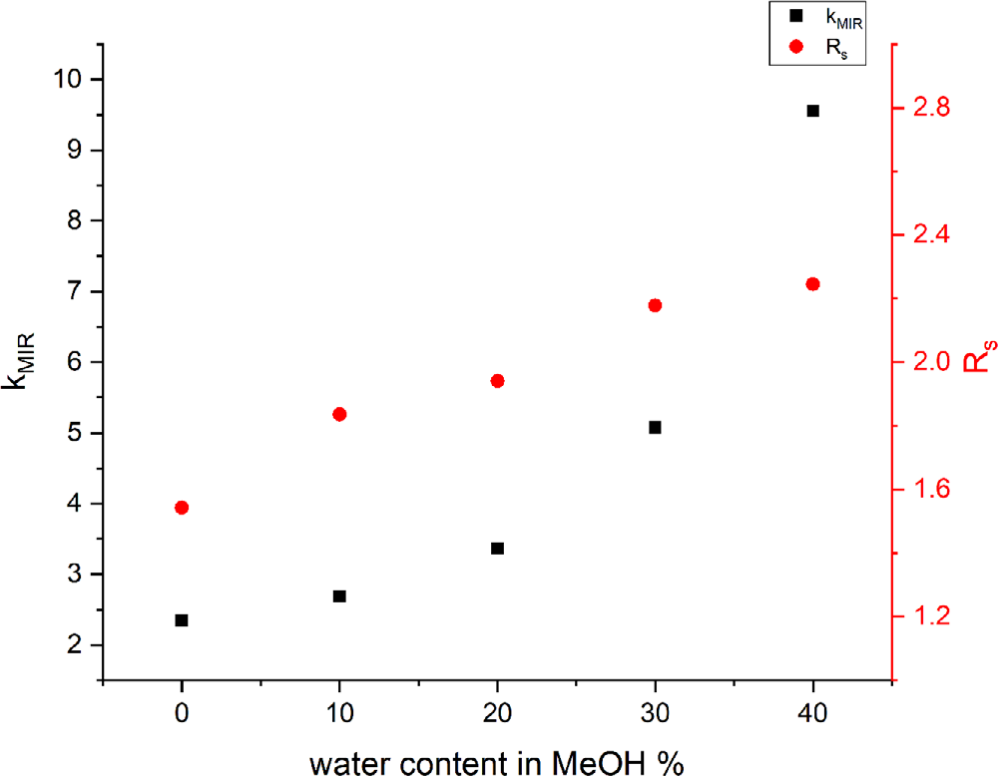
Plots of the retention factor of mirabegron and resolution as a function of the water content in methanol on Chiral CD‐Ph column. Chromatographic conditions: mobile phase 0.1% diethylamine in the indicated eluent composition, flow rate: 0.7 mL/min, column temperature: 25°C.

During the initial screening phase, the performance of seven CD‐based CSPs (Astec Cyclobond I 2000, Quest γ‐CD, Nucleodex β‐PM, Quest C2, Quest CM, Quest SB, Chiral CD‐Ph) was evaluated in both polar organic and reversed‐phase modes. Some representative chromatograms are depicted in Figure S1. Chiral recognition can be observed only on the Chiral CD‐Ph column using MeOH:DEA or MeOH:H_2_O:DEA eluents. The other two columns and interestingly ACN‐based mobile phases did not show successful separation results. Notably, enantiorecognition with baseline separation was achieved only using the Chiral CD‐pH column with a mobile phase composition of MeOH:H_2_O:DEA. As such, all further method developments were performed under these defined conditions. TEA can also be used instead of DEA, but because of the higher UV cut‐off value of TEA, DEA was selected as a basic additive. In the screening phase, the effect of eluent composition on the enantioseparation was also investigated on the Chiral CD‐Ph column using different MeOH:H_2_O:DEA mixtures. Figure 2 illustrates the impact of water content in MeOH in the mobile phase on the retention and enantioresolution of MIR enantiomers when using the Chiral CD‐Ph column. A typical reversed‐phase behavior was observed, where the retention factor increased exponentially with an increase in water content in the mobile phase. The resolution also improved; however, due to significant peak broadening, this improvement became less pronounced at higher water content.

A screening phase with a “one factor at a time” approach was employed to determine which chromatographic parameters influence the enantioseparation the most. The addition of a basic modifier to the eluent was necessary to achieve adequate peak shapes and enantioseparation, however, a concentration higher than 0.1% did not influence the separation significantly. As discussed above, increasing the water content of the mobile phase improves the resolution, but higher than 20% of water in MeOH caused peak broadening and high retention times. Changes in flow rate influenced the resolution in the range studied of 0.6‐0.8 mL/min, also higher flow rates reduced the analysis time. Flow rates above 0.8 mL/min were not employed due to an increase in backpressure. Column temperature was studied in the 20–40°C range and had a significant influence on separation performance. Temperatures above 40°C in accordance with the column specifications were not applied. Further method optimization was performed by a multivariate approach, using experimental design.

### 3.2 Experimental Design‐based Method Optimization

For further method optimization, a full factorial design was employed, where column temperature (between 20°C and 40°C), mobile phase composition (between 80 and 100 MeOH v/v% in water), and flow rate (between 0.6 and 0.8 mL/min) were chosen as input factors. This approach enabled the systematic optimization of the key parameters most influential to the separation process. The response variables selected for optimization were resolution (*R*_s_), the retention time of the later eluting peak (analysis time), and the peak width of the later eluting peak. The experimental matrix and corresponding results are summarized in Table S2. Table S2 represents the original input data with one parallel set, which was randomly mixed to ensure the robustness and reliability of the statistical tests and results.

The quadratic models were analyzed using the ANOVA test. R^2^ values for the different responses are shown in Supplementary Table 3. Based on the results of the ANOVA tests three‐dimensional response surface plots were constructed that represent the relationship between input factors and responses (Figure S2).

**FIGURE 3:**
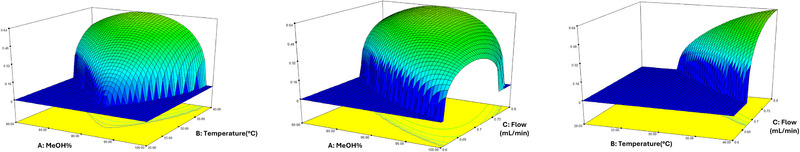
Three‐dimensional response surface plots obtained for desirability.

Our goals were to reach baseline separation between the two enantiomers, have a short analysis time of under 10 min, and minimize peak broadening. Thus, resolution was set to be maximized, and analysis time and peak width were set to be minimized. All three variables had the same weight and relative importance in the design, as all of them are important for good separation performance. To obtain a universal optimum of chromatographic conditions Derringer's desirability function was applied. Figure 3 presents a three‐dimensional response surface plot derived from the regression models.

**FIGURE 4:**
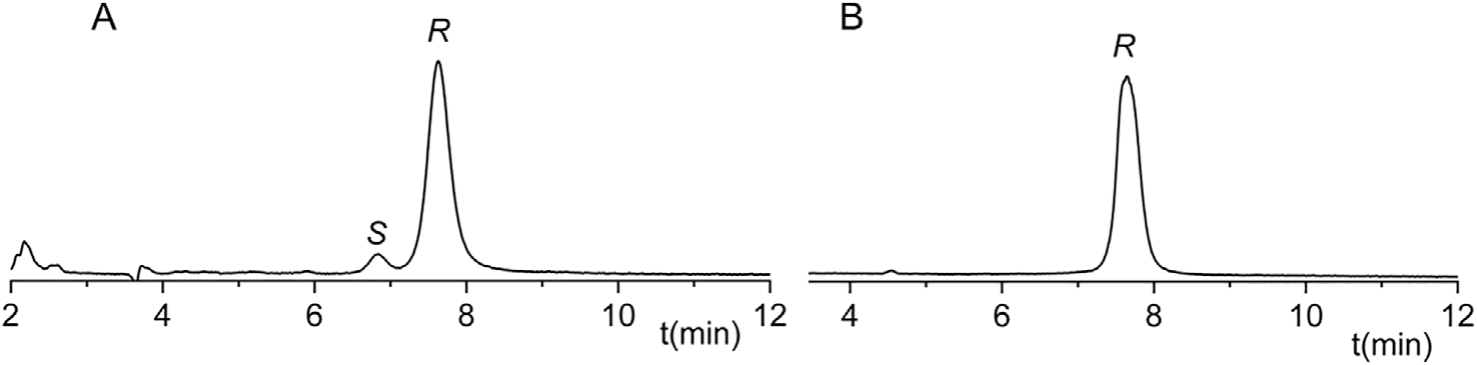
Representative chromatograms obtained during method optimization and application. (A) The Mirabegron sample spiked with 0.1% enantiomeric impurity. (B) Solution of Betmiga 50 mg tablet. Chromatographic conditions: Chiral CD‐Ph column, with MeOH:H_2_O:DEA in a 90:10:0.1 (v/v/v) ratio, flow rate: 0.8 mL/min, column temperature: 40°C.

The optimal analytical conditions, determined through the desirability function, were as follows: Chiral CD‐Ph, mobile phase composition of MeOH:H_2_O:DEA in a 90:10:0.1 (v/v/v) ratio, a flow rate of 0.8 mL/min and a column temperature of 40°C. Under these conditions, baseline separation of the enantiomers was successfully achieved within 10 min using a 2:1 ratio of enantiomers in the injected sample. Using the optimal conditions a 1000:1 ratio of the enantiomers was injected, and method validation was made using this solution. The example chromatogram of the 1000:1 enantiomer ratio sample using optimal circumstances is depicted in Figure 4A.

### 3.3 Method Validation and Application

The method was validated in accordance with The International Council for Harmonization of Technical Requirements for Pharmaceuticals for Human Use (ICH) Q2 (R2) guidelines [30], specifically for the determination of *S*‐MIR as an enantiomeric impurity, with a focus on key parameters such as limit of detection (LOD), limit of quantification (LOQ), linearity, accuracy, and precision. The obtained values are summarized in Table 1.

**TABLE 1:** Summary of data obtained during method validation for the simultaneous determination of enantiomeric purity in a 2 mg/mL 2‐(2‐amino‐1,3‐thiazol‐4‐yl)‐N‐[4‐[2‐[[(2R)‐2‐hydroxy‐2‐phenylethyl]amino]ethyl]phenyl]acetamide (MIR) sample. The S‐MIR concentration was referenced to this concentration.

Parameter	Level	*S*‐MIR
Range (%)		0.04–0.2
Equation		8582.2*x* ‐ 66.702
*r* ^2^		0.9991
LOD (mg/ml)		0.00024
LOQ (mg/ml)		0.0008
Accuracy	I (0.04%)	98.79%
	II (0.14%)	101.22%
	III (0.2%)	100.8%
Intraday precision (RSD%)	I (0.04%)	1.75%
	II (0.14%)	1.19%
	III (0.2%)	0.80%
Intermediate precision (RSD%)	I (0.04%)	1.83%
	II (0.14%)	1.65%
	III (0.2%)	0.90%

The LOD and LOQ values for *S*‐MIR were determined at concentration values yielding a signal‐to‐noise ratio of 3:1, and 10:1, respectively. The LOD was 0.24 µg/mL, while the LOQ was 0.8 µg/mL (corresponding to 0.04% of impurity in the 2 mg/mL MIR sample). The validation of enantiomer determination was performed within the concentration range of 0.04% to 0.2%, relative to a target concentration of 2 mg/mL of MIR. Linearity of the method was confirmed across six concentration levels within this specified range for the enantiomer and calibration plots were represented by plotting peak areas against corresponding concentrations. The calibration plot demonstrated a linear relationship, with correlation coefficients exceeding 0.9991 in all cases. Furthermore, the 95% confidence intervals for the y‐intercepts encompassed zero, and the residuals exhibited a random distribution.

The accuracy and precision were assessed through intra‐day (repeatability) and inter‐day (two consecutive days) evaluations at three concentration levels of the enantiomer, spanning the linearity range. Each solution was injected five times. The accuracy, expressed as a mean recovery percentage, ranged from 98.79% to 101.22% with less than 1% standard deviation. The repeatability expressed as relative standard deviation (RSD%) ranged from 0.80% to 1.75%. The intermediate precision of the method, evaluated over two consecutive days and expressed as RSD% was found to be less than 2%. Based on the obtained results the optimized method demonstrated linearity, accuracy, and precision for the determination of *S*‐MIR as a chiral impurity. The optimized and validated method was applied to analyze a real pharmaceutical sample, specifically a prolonged‐release film‐coated tablet containing a nominal dosage of 50 mg MIR. The representative chromatogram of the pharmaceutical formulation is shown in Figure 4B. The application of the developed method revealed that the commercial formulation does not contain a detectable amount of chiral impurity.

### 3.4 Mechanism of Chiral Recognition

The investigation of chiral recognition mechanisms is crucial for understanding the driving forces of chiral separation. Among the various techniques available for this purpose, thermodynamic analysis and docking studies were applied to gain deeper insights into the enantioselective interactions between MIR and chiral selectors [31].

### 3.5 Thermodynamic Analysis

Thermodynamic analysis is a valuable and widely used approach for investigating chiral recognition mechanisms [32, 33, 34]. In this study, chromatographic runs conducted at varying temperatures (20, 25, 30, 35, and 40°C) enabled the comparison of thermodynamic parameters for the separation of MIR enantiomers in both reversed‐phase and polar organic modes. To assess the impact of temperature on retention and selectivity, the classical van’ t Hoff analysis was employed. While this method is favored for its simplicity, it does not differentiate between enantioselective and non‐enantioselective interactions, rendering the derived thermodynamic parameters apparent rather than absolute. This means that in chiral chromatography, the conclusions drawn from van't Hoff plots can be misleading because several different types of adsorption sites may be present on the surface of the stationary phase [35, 36].

Based on the classical van’ t Hoff method the differences in standard enthalpy Δ(Δ*H*°) and standard entropy Δ(Δ*S*°) for the enantiomeric pair in reversed‐phase and polar organic modes on the Chiral CD‐Ph column were determined by plotting lnα *vs*. 1/T as follows:

(1) $$ln\alpha =-\frac{\Delta \left(\Delta H^{\circ }\right)}{RT}+\frac{\Delta \left(\Delta S^{\circ }\right)}{R}$$

where *R* stands for universal gas constant, *T* is the temperature expressed in Kelvin and α is the selectivity factor.

Besides these parameters, isoenantioselective temperatures (*T*_iso_) were calculated as the ratio of Δ(Δ*H*°) to Δ(Δ*S*°). *T*_iso_ represents the temperature at which the enthalpic and entropic contributions cancel each other out, resulting in a Gibbs free energy difference Δ(Δ*G*°) of zero. At this temperature, the two enantiomers co‐elute and no separation occurs. The value of Δ(Δ*G*°) provides insight into the strength of binding between the selector and selectant, with more negative values indicating stronger, more efficient binding. Therefore, when Δ(Δ*G°*) = 0, there is no difference in the binding strength of the enantiomers, leading to co‐elution and a lack of separation at *T*_iso_. The obtained thermodynamic data using different mobile phase compositions using 0.6 mL/min flow rate are summarized in Table 2.

**TABLE 2:** Calculated thermodynamic parameters on Chiral CD‐Ph column (mean ± SD).

Water content (%)	Equation	*r* ^2^	Δ(Δ)H° (kJ/mol)	Δ(Δ) S° (J/mol K)	Δ(Δ) G° (kJ/mol)	T_iso_ (°C)	Q^*^
0	*y* = 238.47*x* ‐ 0.624	0.9978	−1.98 ± 0.10	−5.19 ± 0.15	−0.44 ± 0.06	109.3 ± 8.5	1.28 ± 0.03
5	*y* = 257.26*x* ‐ 0.667	0.9993	−2.14 ± 0.12	−5.54 ± 0.14	−0.49 ± 0.08	112.9 ± 12.2	1.30 ± 0.04
10	*y* = 271.92*x* ‐ 0.716	0.9987	−2.26 ± 0.06	−5.95 ± 0.08	−0.49 ± 0.04	106.9 ± 5.04	1.27 ± 0.02
20	*y* = 290.22*x* ‐ 0.781	0.9994	−2.41 ± 0.07	−6.49 ± 0.05	−0.48 ± 0.05	98.46 ± 8.00	1.25 ± 0.03

* Q = Δ(ΔH°)/(T* Δ(Δ)S°) 298K.

By analyzing the thermodynamic data, it can be observed that upon the addition of water Δ(Δ)*H*°, Δ(Δ)*S*° values decrease, while Δ(Δ)*G*° does not change significantly. The more negative Δ(Δ)*H*° value in terms of water content suggests that the interaction becomes more exothermic with higher water content, which could be due to enhanced solvation effects, while the more negative Δ(Δ)*S*° value indicates a decrease in system randomness, that can easily interpret with the stronger binding interaction. The *T_i_*_so_ value is near 100°C regardless of the mobile phase used, and the Q value is higher than 1.25, indicating that the enantioseparation is enthalpy controlled.

### 3.6 Docking Study

**FIGURE 5:**
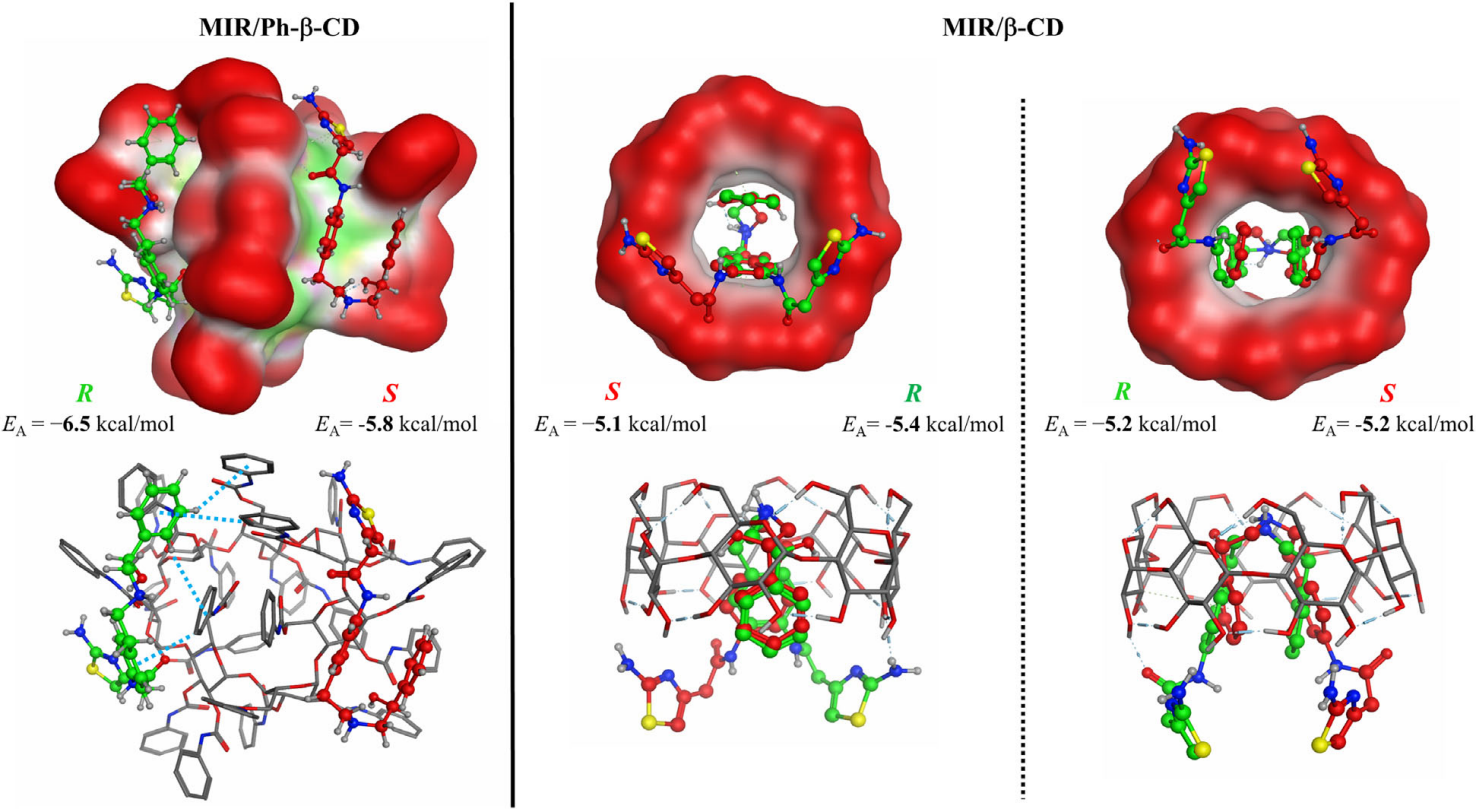
Binding modes and affinities of mirabegron enantiomers toward phenylcarbamate‐β‐CD and β‐CD. The molecular surface of the hosts is also visualized, where exposed regions (to the solvent), hydrophobic groups, and polar groups are represented by red, green, and magenta, respectively. The identified π–π interactions with the host in the case of the R‐mirabegron/phenylcarbamate‐β‐CD complex are represented with light blue dashed lines.

To model the enantioseparation process and chiral recognition of MIR enantiomers on the Chiral CD‐Ph column, and β‐CD column, molecular docking calculations were carried out. The interactions established between the MIR enantiomers and the modeled hosts, phenylcarbamate‐β‐CD and β‐CD, were determined and the most feasible complexes were selected and compared (Figure 5).

The MIR enantiomers interact with the surface of the phenylcarbamate‐β‐CD since the phenylcarbamate groups are blocking the cavity of the host. Thus, surface binding was established in both *R*‐ and *S*‐MIR/phenylcarbamate‐β‐CD complexes (Figure 5, left). However, in the case of β‐CD, the cavity is accessible, and the ligands form inclusion complexes with the host (Figure 5, middle and right). Based on modeling results, two very similar pairs of structures are possible with comparable binding affinities in the case of β‐CD MIR complex. According to the binding affinities, *R*‐MIR displays a stronger interaction with the host (−6.5 kcal/mol) compared to the *S* enantiomer (−5.8 kcal/mol) in the case of the phenylcarbamate‐β‐CD. The difference (Δ*E*_A_ = 0.7 kcal/mol) is large enough to indicate that the separation is possible, and it aligns well with experimental findings. The thiazole part of the enantiomers is involved in interactions with the CD, but no huge differences can be identified. However, the *R*‐MIR established at least four π–π interactions with the host, while these are missing in the case of the *S*‐MIR/phenylcarbamate‐β‐CD surface complex (Figure 5, left). Potentially, this structural discrepancy is responsible for the underlying energetical differences of the complexes.

In the case of the first pair of MIR/β‐CD complexes, the difference in the binding affinities is smaller, only Δ*E*_A_ = 0.3 kcal/mol but still favoring the *R* enantiomer (Figure 5, middle). From a structural point of view, the thiazole group of the enantiomers is outside of the cavity and establishes interaction with the rim of the CD. A similar pattern can be seen in the case of the other pair of β‐CD complexes. Only the orientation of the *R‐* and *S*‐MIR enantiomers differ. In this case, the difference between the binding affinities is zero (Figure 5, right), which shows that the enantioseparation of MIR with β‐CD is not feasible. The modeling confirms the experimental observation that attaching an aromatic ring to the CD core structure is necessary for enantiorecognition.

## 4 Conclusion

A robust and efficient HPLC method was developed for the enantioseparation of MIR and its chiral impurity, *S*‐MIR, using CD‐based CSPs. The initial phase of method development involved evaluating the chiral discrimination capabilities of seven CD‐derived columns under both polar organic and reversed‐phase modes. Among these, the Chiral CD‐Ph column, utilizing phenylcarbamate‐modified β‐CD, demonstrated superior performance in achieving enantiomeric resolution. Multivariate optimization of chromatographic conditions, including the mobile phase composition, column temperature, and flow rate led to the identification of the following optimal chromatographic parameters: a mobile phase of 90:10:0.1 MeOH:H_2_O:DEA, a flow rate of 0.8 mL/min, and a column temperature of 40°C. The method was validated in accordance with ICH guidelines. The validated method was successfully applied to real sample analyses, demonstrating its potential for ensuring the enantiomeric purity of MIR and fulfilling regulatory requirements for impurity quantification. Additionally, thermodynamic methods and molecular modeling were used to investigate the processes leading to enantiorecognition, revealing that the differing binding of MIR enantiomers to the surface of the chiral selector is responsible for enantiorecognition. The developed method offers a fast and reliable approach for the enantioseparation and analysis of MIR, supporting its safe and effective use in pharmaceutical applications.

## Supporting information



Supporting Information
