# Supplementary material for: Enantioseparation of Mirabegron Using Cyclodextrin‐based Chiral Columns: High‐performance Liquid Chromatography and Molecular Modeling Study
Source: J Sep Sci. 2025 Apr 9;48(4):e70132. doi: 10.1002/jssc.70132 (PMC11982617; doi:10.1002/jssc.70132)
Supplement: Supplementary file 1 — Supporting Information [file JSSC-48-e70132-s001.docx]

**SUPPLEMENTARY MATERIALS TO**

**Enantioseparation of mirabegron using cyclodextrin-based chiral columns: HPLC and molecular modeling study**

Ali Mhammad^1,2^, Gergely Dombi^1,2^, Máté Dobó^1,2^, Zoltán-István Szabó^3,4^, Béla Fiser^5,6,7^, Gergő Tóth^1,2 *^

^1^ Department of Pharmaceutical Chemistry, Semmelweis University, Hőgyes E. u. 9, Budapest, 1092, Hungary

^2^ Center for Pharmacology and Drug Research & Development, Semmelweis University, Budapest, Hungary

^3^ George Emil Palade University of Medicine, Pharmacy, Science, and Technology of Targu Mures, Târgu Mureş, Romania

^4^ Sz-imfidum Ltd, Lunga, 525401, Romania

^5^ Institute of Chemistry, University of Miskolc, 3515 Miskolc, Hungary

^6^ Department of Biology and Chemistry, Ferenc Rákóczi II Transcarpathian Hungarian College of Higher Education, Transcarpathia, 90200 Beregszász, Ukraine

^7^ Department of Physical Chemistry, Faculty of Chemistry, University of Lodz, 90-149 Łódź, Poland

* Corresponding author: [toth.gergo@semmelweis.hu](mailto:toth.gergo@semmelweis.hu)

Supplementary Table 1. Different mobile phase compositions used in the preliminary study

| Mobile Phase 1 (MeOH:H_2_O:DEA) | Mobile Phase 2 (ACN:H_2_O:DEA) |
| --- | --- |
| 100:0:0.1 | 100:0:0.1 |
| 90:10:0.1 | 90:10:0.1 |
| 80:20:0.1 | 80:20:0.1 |
| 70:30:0.1 | 70:30:0.1 |
| 60:40:0.1 | 60:40:0.1 |

Supplementary Table 2. Full factorial design table used for method optimization with the obtained results for the selected responses.

| Run No. | MeOH in Water (%) * (Factor 1) | Temperature (°C) (Factor 2) | Flow rate (mL/min) (Factor 3) | Analysis Time (min) | Peak width  (min) | R_s_ |
| --- | --- | --- | --- | --- | --- | --- |
| 1 | 90 | 30 | 0.6 | 12.34 | 0.523 | 2.2 |
| 2 | 100 | 20 | 0.6 | 12.815 | 0.847 | 1.6 |
| 3 | 80 | 35 | 0.8 | 9.976 | 0.411 | 2 |
| 4 | 90 | 40 | 0.7 | 9.327 | 0.361 | 2.1 |
| 5 | 95 | 40 | 0.6 | 10.711 | 0.411 | 2.1 |
| 6 | 100 | 40 | 0.8 | 7.287 | 0.32 | 1.8 |
| 7 | 95 | 35 | 0.8 | 8.346 | 0.402 | 1.9 |
| 8 | 80 | 25 | 0.7 | 13.452 | 0.649 | 2.1 |
| 9 | 90 | 40 | 0.6 | 10.926 | 0.399 | 2.2 |
| 10 | 95 | 25 | 0.8 | 9.59 | 0.567 | 1.7 |
| 11 | 100 | 30 | 0.7 | 9.597 | 0.51 | 1.8 |
| 12 | 95 | 30 | 0.6 | 12.128 | 0.578 | 2 |
| 13 | 80 | 20 | 0.6 | 17.385 | 0.878 | 2 |
| 14 | 90 | 35 | 0.6 | 11.641 | 0.448 | 2.2 |
| 15 | 95 | 25 | 0.6 | 12.959 | 0.668 | 1.9 |
| 16 | 100 | 35 | 0.6 | 10.54 | 0.475 | 1.8 |
| 17 | 90 | 35 | 0.7 | 9.93 | 0.414 | 2.1 |
| 18 | 80 | 30 | 0.6 | 14.491 | 0.581 | 2.2 |
| 19 | 95 | 30 | 0.7 | 10.221 | 0.515 | 1.9 |
| 20 | 90 | 30 | 0.8 | 9.123 | 0.448 | 1.9 |
| 21 | 95 | 40 | 0.7 | 9.061 | 0.368 | 2 |
| 22 | 80 | 35 | 0.7 | 11.492 | 0.44 | 2.1 |
| 23 | 100 | 25 | 0.8 | 8.955 | 0.586 | 1.6 |
| 24 | 80 | 20 | 0.7 | 14.766 | 0.811 | 2 |
| 25 | 90 | 20 | 0.8 | 10.599 | 0.627 | 1.8 |
| 26 | 90 | 20 | 0.7 | 12.151 | 0.678 | 1.9 |
| 27 | 100 | 35 | 0.8 | 7.765 | 0.385 | 1.8 |
| 28 | 80 | 40 | 0.8 | 9.153 | 0.349 | 2 |
| 29 | 100 | 20 | 0.7 | 10.955 | 0.76 | 1.5 |
| 30 | 95 | 40 | 0.8 | 7.915 | 0.324 | 1.9 |
| 31 | 95 | 20 | 0.7 | 11.73 | 0.7 | 1.7 |
| 32 | 90 | 25 | 0.7 | 11.393 | 0.582 | 2 |
| 33 | 80 | 40 | 0.6 | 12.578 | 0.433 | 2.2 |
| 34 | 95 | 30 | 0.8 | 8.877 | 0.477 | 1.8 |
| 35 | 80 | 25 | 0.6 | 15.866 | 0.723 | 2.2 |
| 36 | 100 | 40 | 0.6 | 9.939 | 0.412 | 2 |
| 37 | 100 | 20 | 0.8 | 9.551 | 0.71 | 1.4 |
| 38 | 100 | 25 | 0.6 | 12.073 | 0.718 | 1.7 |
| 39 | 100 | 30 | 0.8 | 8.323 | 0.461 | 1.7 |
| 40 | 95 | 35 | 0.6 | 11.345 | 0.451 | 2.1 |
| 41 | 95 | 35 | 0.7 | 9.55 | 0.416 | 2 |
| 42 | 80 | 35 | 0.6 | 13.428 | 0.497 | 2.3 |
| 43 | 100 | 30 | 0.6 | 11.27 | 0.586 | 1.8 |
| 44 | 80 | 30 | 0.8 | 10.703 | 0.476 | 2.1 |
| 45 | 90 | 40 | 0.8 | 8.06 | 0.327 | 1.9 |
| 46 | 80 | 40 | 0.7 | 10.683 | 0.394 | 2.1 |
| 47 | 95 | 20 | 0.8 | 10.23 | 0.676 | 1.6 |
| 48 | 90 | 20 | 0.6 | 14.284 | 0.751 | 2 |
| 49 | 95 | 25 | 0.7 | 11.019 | 0.619 | 1.8 |
| 50 | 80 | 25 | 0.8 | 11.716 | 0.593 | 2 |
| 51 | 90 | 25 | 0.6 | 13.39 | 0.636 | 2.1 |
| 52 | 80 | 20 | 0.8 | 12.896 | 0.751 | 1.9 |
| 53 | 90 | 30 | 0.7 | 10.476 | 0.469 | 2 |
| 54 | 100 | 40 | 0.7 | 8.459 | 0.354 | 1.8 |
| 55 | 80 | 30 | 0.7 | 12.304 | 0.522 | 2.1 |
| 56 | 95 | 20 | 0.6 | 13.956 | 0.791 | 1.8 |
| 57 | 90 | 35 | 0.8 | 8.67 | 0.397 | 2 |
| 58 | 100 | 35 | 0.7 | 8.955 | 0.412 | 1.8 |
| 59 | 100 | 25 | 0.7 | 10.33 | 0.64 | 1.7 |
| 60 | 90 | 25 | 0.8 | 9.833 | 0.524 | 1.8 |

Supplementary Table 3**.** Summary of R-squared (R^2^), adjusted R-squared (Adj. R^2^) and predicted R-squared (Pred. R^2^) values for the different responses

| Analyzed response | R^2^ | Adj. R^2^ | Pred R^2^ |
| --- | --- | --- | --- |
| Analysis time | 0.9941 | 0.9930 | 0.9912 |
| Peak width of last eluting peak | 0.9815 | 0.9782 | 0.9723 |
| Resolution | 0.9556 | 0.9476 | 0.9326 |

**
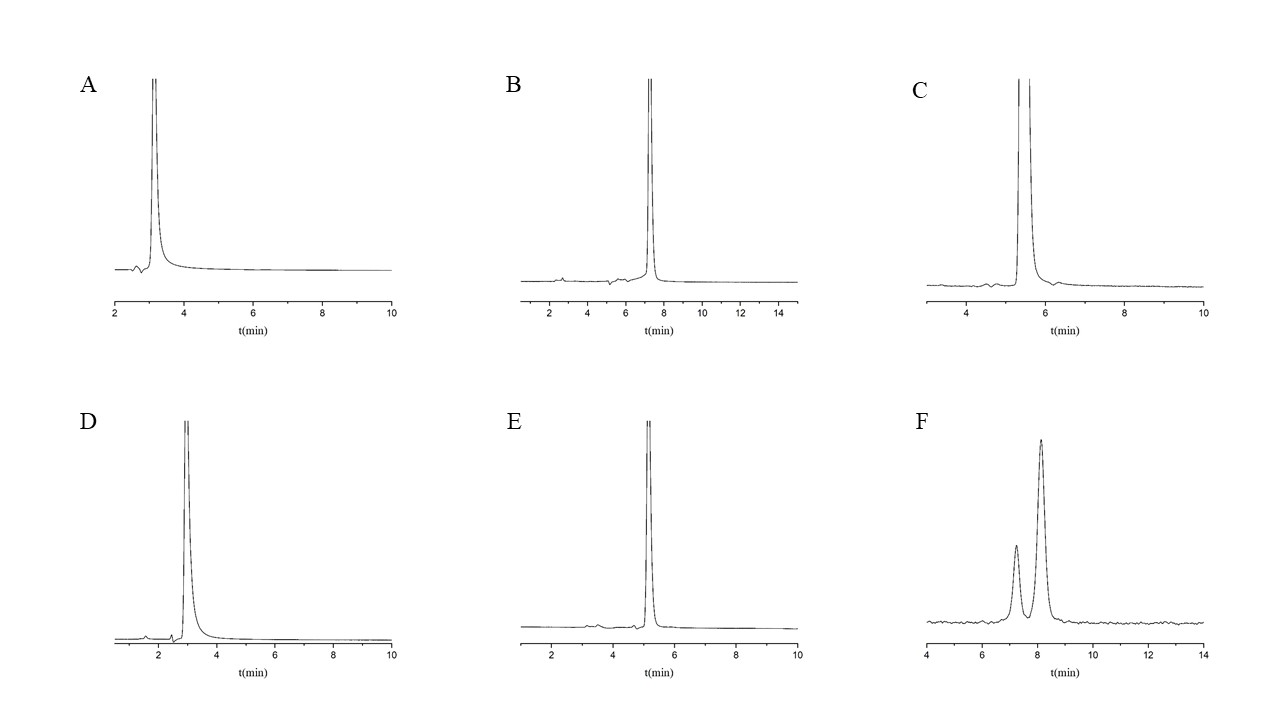
**

**Supplementary Figure 1.** Chromatograms from screening phase A) Nucleodex β-PM with ACN:H_2_O:DEA 90:10:0.1; B) Cyclobond I 2000 with ACN:H_2_O:DEA 90:10:0.1; C) Chiral CD-Ph with ACN: H_2_O: DEA 90:10:0.1; D) Nucleodex β-PM MeOH: H_2_O: DEA 90:10:0.1; E) Cyclobond I 2000 MeOH: H_2_O: DEA 90:10:0.1; F) Chiral CD-Ph with MeOH: H_2_O: DEA 90:10:0.1. The flow rate was uniformly 0.7 mL/min, column temperature was 25 °C. Chiral separation was observed only in Chiral CD-Ph column.

**
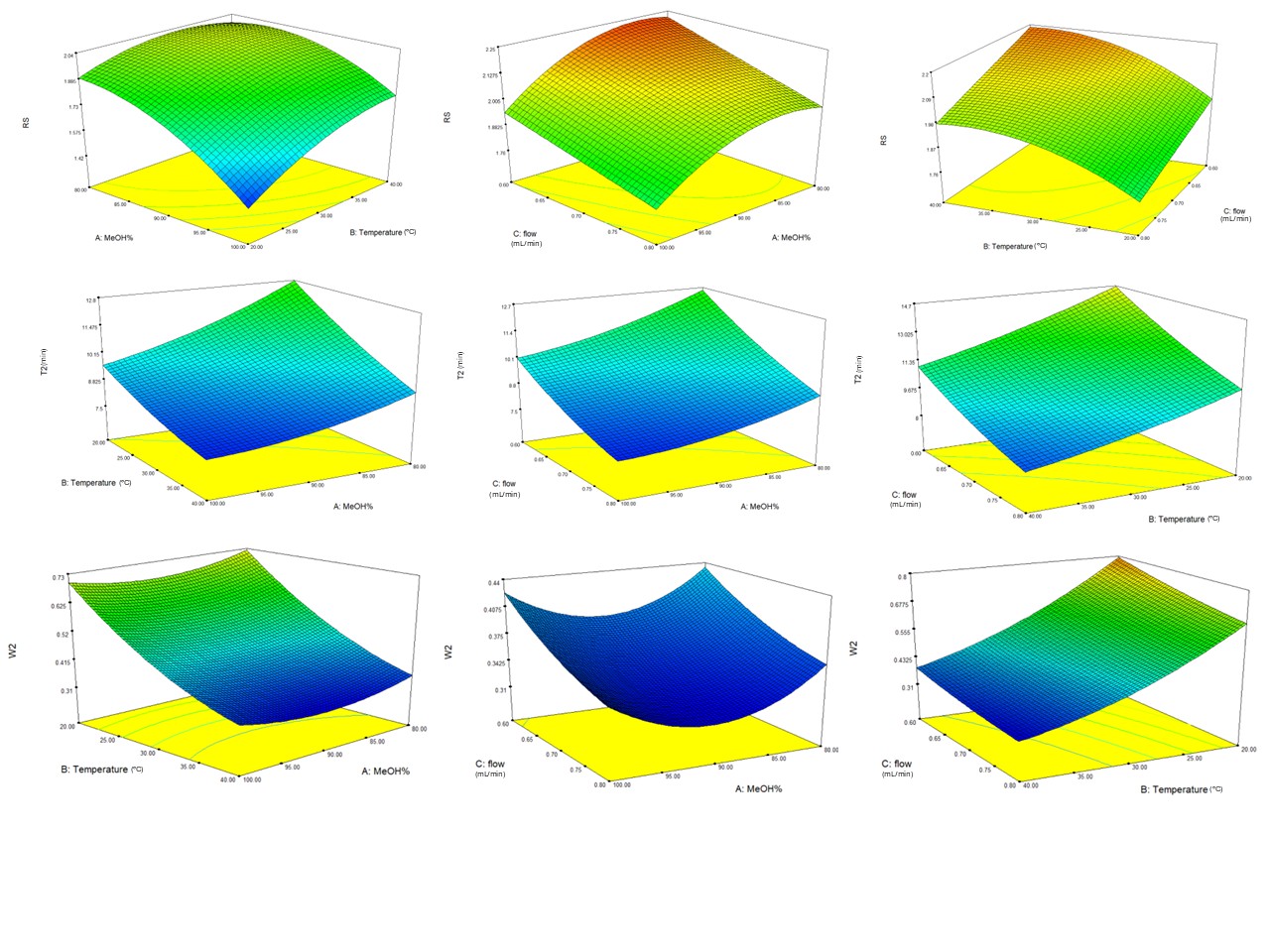
**

**Supplementary Figure 2.** Three-dimensional response surface plots between the input factors and the specific responses. First line – resolution, second line – analysis time, third line – peak width. One input factor is always kept at constant, the used constant value is chosen by the results of the design, and the most desirable condition is used.
